# Marine pharmaceutical monitoring with POCIS: sampling rate uncertainty, and implications for regulation

**DOI:** 10.1007/s10661-026-15742-y

**Published:** 2026-08-08

**Authors:** Heidi Ahkola, Lauri Äystö, Mailis Laht, Vallo Kõrgmaa, Jukka Mehtonen, Kai Myrberg, Ljudmila Vesikko, Ville Junttila

**Affiliations:** 1 Finnish Environment Institute (Syke), Latokartanonkaari 11, 00790 Helsinki, Finland; 2 Estonian Environmental Research Centre, Marja 4D, 10617 Tallinn, Estonia

**Keywords:** Passive sampling, Sampling rate, Polar organic chemical integrative sampler, POCIS, Pharmaceuticals

## Abstract

POCIS passive samplers were deployed at differently polluted marine sites in the cities of Helsinki and Tallinn and in an open part of the Gulf of Finland. The aim was to determine baseline concentration levels for pharmaceuticals in water monitored with passive sampling techniques, which could also be applied to regulatory monitoring. That baseline concentration data method could then be applied to other sites in the Tallinn and Helsinki area to assess whether the pharmaceutical concentration is elevated or at the baseline level. Altogether, 150 pharmaceuticals were analysed from passive and grab water samples, and 30 substances were discovered. The number of pharmaceuticals detected in passive samplers (27) was higher than in grab samples (20). However, the study revealed the presence of several pharmaceuticals even from expected background sites, such as the open sea site in Gulf of Finland, implying their ubiquitous presence in the study area. The passive sampling results have been converted to TWAC with sampling rate, which is characteristic of the sampler type and studied compound. We assessed the suitability and differences of sampling rates collected from the literature, calculated from LogK_OW_ values and estimated from field deployment results. The selection of sampling rate is essential, as it can cause 86 to 99% differences in determined TWACs. The TWAC was compared with PNEC was calculated to evaluate the potential environmental risk. There are still open questions regarding how to reconcile the passive sampling results with regulatory monitoring obligations based on grab water sampling and existing limit concentrations.

**Supplementary Information:**

The online version contains supplementary material available at https://doi.org/10.1007/s10661-026-15742-y.

## Introduction

Pharmaceuticals in the environment may have adverse effects on organisms even at very low concentrations. Although most pharmaceuticals are not persistent, they are constantly present in the environment due to their continuous release from, for instance, wastewater treatment plants (WWTPs). Oceans act as a sink for pharmaceutical loading (Chen et al., 2025; Zandaryaa & Frank-Kamenetsky, 2021). However, low but biologically significant concentrations may be challenging to detect using only traditional sampling methods.

Grab water sampling is used in the regulatory monitoring of harmful chemicals. However, its limitations have been recognised, as it provides only a snapshot of the chemical concentration at the time of sampling (Röemerscheid et al., 2023; Taylor et al., 2021). Environmental concentrations of pharmaceuticals have been demonstrated to vary depending on both environmental conditions and seasonal patterns of use (Baumgartner et al., 2024; Lindholm-Lehto et al., 2016; Vieno et al., 2005). As the concentrations fluctuate, the timing of the sampling event affects whether the concentration peak is detected or remains undiscovered. Hence, the concentration below the detection limit does not automatically mean that the chemical is not present at the study site. Therefore, an instant grab water sample cannot necessarily be considered representative of the chemical status of the watercourse. With passive sampling, the chemicals are accumulated into the sampler material over a time period of up to several weeks. This can help to enrich the trace concentrations, which remain undetected in grab water samples, to a measurable level. Thus, passive sampling can bring new insights to discussions about monitoring harmful chemicals.

The Polar Organic Chemical Integrative Sampler (POCIS) is widely used and is the first commercial passive sampler for studying moderately polar to polar organic chemicals (Alvarez et al., 2004; Bayen et al., 2014; Vrana et al., 2021). The most frequently used receiving phase of the POCIS sampler is the hydrophilic lipophilic balance (HLB) material sandwiched between two polyethersulfone (PES) membranes. For instance, Poulier et al. (2015) monitored pesticides with POCIS samplers and grab water samples and automatically collected composite water samplers and noticed that POCIS detected trace concentrations and yielded more representative data of studied compounds which were not even detected in water samples.

In this study, POCIS passive samplers were deployed in Finnish and Estonian coastal waters in the Gulf of Finland to study the presence of pharmaceuticals in the autumn of 2023 and the spring and summer of 2024. Time-weighted average concentrations (TWAC) were calculated with sampling rates obtained from the literature, by deriving from the LogK_OW_ value and determining in situ field sampling results; additionally, the effect of different sampling rates on the concentrations is discussed. The aim was to determine baseline concentration levels for pharmaceuticals monitored with passive sampling techniques and to apply that to selected locations in the Tallinn and Helsinki coastal area to assess whether the pharmaceutical concentration is elevated or at the baseline level.

## Materials and methods

### Study area

The study area, the Gulf of Finland, is located at the north-eastern extremity of the Baltic Sea. The gulf is an elongated estuary-like water body (length c. 400 km, width from 48 to 135 km) with a mean depth of only 37 m and an extremely complex coastline. The western end of the gulf is a direct continuation of the Baltic Sea Proper, whereas the eastern end receives the largest single freshwater inflow to the Baltic Sea from the discharge of the River Neva (Myrberg & Soomere, 2013). The Estonian coast is rather deep (60—100 m) and has more or less regular bathymetry, with only a few islands, whereas the Finnish coast is shallow (20—40 m), has an extremely irregular coastline and ragged bathymetry and contains extensive archipelago areas (Leppäranta and Myrberg, 2009).

**Fig. 1:**
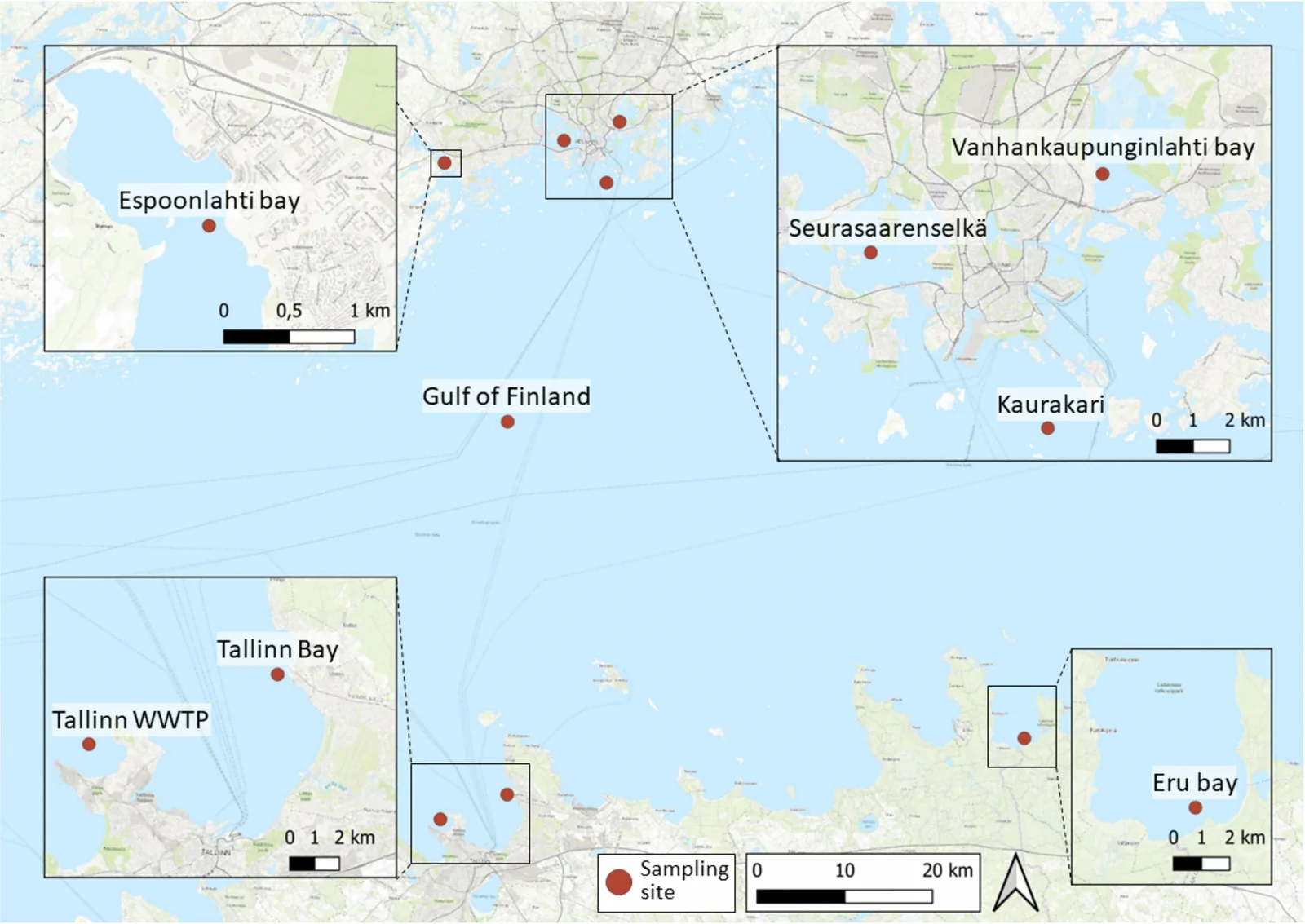
Location of sampling sites. Basemap: ESRI World Topo

POCIS passive samplers were deployed at eight sampling sites, four of which were located in the Finnish coastal area (around the City of Helsinki), three in the Estonian costal area and one in an open part of the Gulf of Finland (Fig. 1). The selected locations included sites with expected low and high pharmaceutical concentrations, such as sites near wastewater treatment plants (Tallinn WWTP) and the Vanhankaupunginlahti Bay, which receives discharges from WWTP effluents released to the River Vantaanjoki. The sites at Seurasaarenselkä, Espoonlahti bay, Kaurakari and Tallinn Bay receive diffuse pollution, and Kaurakari is also affected to some extent by the River Vantaanjoki flowing to Vanhankaupunginlahti Bay. Eru Bay, located in the Lahemaa National Park, has no direct human impact from the catchment area.

### Sampling and analysis

POCIS passive sampler has 200 mg of HLB sorbent granules sandwiched between two PES membranes. The samplers and deployment cages were purchased from Affinisep, France. POCIS passive samplers were deployed on 16 sampling occasions at eight sampling sites (Table 1). One to two samplers were attached to the stainless-steel sampler cages. Both samplers from the first deployment in November 2023 were analysed for pharmaceutical concentrations. During subsequent deployments, the second sampler was stored for potential future use. The deployment time varied between 21 and 35 days; the exception was the sampling conducted in the open sea in the Gulf of Finland, which lasted for 55 days due to R/V Aranda’s time schedule. In coastal sites, the deployment depth was targeted at half of the water column depth, resulting in depths ranging from 0.8 to 6 m. In the 30-m-deep open sea site (the Gulf of Finland), the deployment depth was 10 m (Table 1).

**Table 1:** Information about the passive and grab water sampling in the Gulf of Finland

POCIS passive sampling					
Site	Beginning of deployment (dd.mm.yyyy)	End of deployment (dd.mm.yyyy)	Deployment time (d)	Sampling depth (m)	Number of detected substances (number of quantified)/number of analysed
Vanhankaupunginlahti Bay	25.04.2024	30.05.2024	35	0.8	24 (22)/147
Vanhankaupunginlahti Bay	30.05.2024	02.07.2024	33	0.8	23 (19)/147
Seurasaarenselkä	09.10.2023	06.11.2023	28	3.0	15 (7)/149
Seurasaarenselkä	25.04.2024	30.05.2024	35	3.0	13 (11)/147
Seurasaarenselkä	30.05.2024	02.07.2024	33	3.0	12 (8)/147
Espoonlahti Bay	09.10.2023	06.11.2023	28	3.0	17 (8)/149
Kaurakari	25.04.2024	30.05.2024	35	3.0	14 (10)/147
Kaurakari	30.05.2024	02.07.2024	33	3.0	13 (10)/147
Open sea	15.04.2024	09.06.2024	55	10	8 (6)/150
Eru Bay	01.11.2023	22.11.2023	21	1.0	8 (2)/149
Eru Bay	07.05.2024	05.06.2024	29	1.0	8 (4)/147
Eru Bay	05.06.2024	01.07.2024	26	1.0	8 (6)/147
Tallinn WWTP	09.05.2024	06.06.2024	28	2.0	11 (8)/147
Tallinn Bay	03.11.2023	24.11.2023	21	6.0	11 (5)/149
Tallinn Bay	09.05.2024	06.06.2024	28	6.0	10 (7)/147
Tallinn Bay	06.06.2024	02.07.2024	26	6.0	5 (4)/147
Grab water sampling					
Site	**Sampling date (dd.mm.yyyy)**			**Sampling depth (m)**	**Number of detected substances/number of analysed**
Vanhankaupunginlahti Bay	25.4.2024			0.8	15/149
Vanhankaupunginlahti Bay	30.5.2024			0.8	19/149
Vanhankaupunginlahti Bay	2.7.2024			0.8	13/149
Seurasaarenselkä	25.4.2024			3.0	4/149
Seurasaarenselkä	30.5.2024			3.0	2/149
Seurasaarenselkä	2.7.2024			3.0	1/149
Kaurakari	25.4.2024			3.0	3/149
Kaurakari	30.5.2024			3.0	1/149
Kaurakari	2.7.2024			3.0	1/149

After retrieval, the HLB granules from the POCIS samplers were extracted twice with 15 mL methanol and were analysed with high-performance liquid chromatography combined with tandem mass spectrometry (HPLC/MS/MS), according to USEPA (2007). In this study, 147–149 substances were analysed altogether, and the list of analysed substances in passive and grab water samples and their quantification limits (LOQ) are presented in Tables S1-S2.

To assess the differences between passive and grab water sampling, grab water samples were taken from three passive sampling sites (Vanhankaupunginlahti Bay, Seurasaarenselkä and Kaurakari) from the same depth samplers were deployed in the summer of 2024. Two passive sampling deployments were conducted, and the grab water samples were taken as the passive samplers were replaced with new ones in the beginning and in the end of each deployment. Grab water samples were taken to clean glass bottles provided by the analysing laboratory; they were then solid phase extracted (SPE) with HLB sorbent and further analysed as POCIS passive sampler extracts (USEPA, 2007). It should be noted that TWAC describes the dissolved chemical fraction; thus, it differs from the total chemical concentration determined in grab water samples.

### Determination of TWAC

The amount of substance in the POCIS passive sampler can be converted to TWAC during the deployment time C_W_ (ng L^−1^) using Eq. 1:

1 $${C}_{W}=\frac{{C}_{S}{M}_{S}}{{R}_{S}t}$$

where C_S_ is the concentration of pharmaceutical in POCIS sampler receiving phase (ng g^−1^), M_S_ is the mass of the sorbent according to the manufacturer (200 mg), R_S_ the sampling rate (L d^−1^) and t is the deployment time in days (d) (Alvarez et al., 2004). The accumulation of selected pharmaceuticals to POCIS with the HLB receiving phase has been shown to remain linear over at least 56 days’ deployment time at a concentration of 1 µg L^−1^. In this study, POCIS is considered to act as an infinite sink and the uptake of chemicals to remain linear during the deployment time. However, shorter linear sampling periods such as 4–14 days have been reported in e.g., WWTPs (Domínguez-García et al., 2025; Harman et al., 2011).

### Sampling rates

#### Literature sampling rates

Sampling rates published in the literature (R_Literature_) are generally determined in laboratory calibration tests. The published sampling rates are mainly compound-and-exposure-specific approximations (Harman et al., 2012). Literature sampling rates were not available for all detected substances and POCIS samplers with the HLB receiving phase. Additionally, the receiving phase material affects the sampling rate; thus, sampling rates for other POCIS receiving phase materials were not included (Booij & Chen, 2018). Only sampling rates determined in surface water or laboratory conditions were included and e.g. wastewater calibrations were excluded due to different environmental conditions. Sampling rates published in literature are mainly determined in controlled laboratory conditions using ultrapure water, which often differ from the environmental conditions prevailing at field exposure. Sampling rates are affected by, for instance, water flow rate, temperature, salinity, pH and biofouling (Lotufo et al., 2018; Vrana et al., 2021).

#### Generic sampling rate

As the properties of studied chemicals and the effect of environmental variables cannot be fully controlled, the selection of a single unbiased sampling rate for all compounds is not possible, being always an estimation. The generic sampling rate can provide TWAC estimations for compounds with unknown uptake kinetics (Harman et al., 2012; Röemerscheid et al., 2023). Harman et al. (2012) collected literature sampling rates for different harmful substances using the POCIS passive sampler. The reported sampling rates were determined with different calibration methods and suggested that compounds with a low sampling rate had a low affinity to the POCIS sampler receiving phase. Additionally, high sampling rates may be erroneous, as other studies for the same compounds (e.g. triclosan, fluoxetine, omeprazole) have indicated lower sampling rates. They observed that the median sampling rate values for stagnant and turbulent conditions were 0.18 L day^−1^ and 0.19 L day^−1^, respectively. Tousová et al. (2019) used an estimation of 0.2 L day^−1^ as a generic sampling rate for pharmaceuticals and pesticides. This semi-quantitative technique is suitable for screening and identifying different areas and helps to address further studies. A generic sampling rate of R_Generic_ = 0.18 L d^−1^ was selected to be used in this study, as the water flow at sampling sites was low.

#### Sampling rates calculated from logK_OW_

Sampling rates can also be calculated from K_OW_ according to Eq. 2 (Martínez-Megías et al., 2024; Rico et al., 2019):

2 $${R}_{Eq.2}=0.08+0.02\, log{K}_{OW}$$

LogK_ow_ values were collected for the analytes from PubChem (Kim et al., 2025) using the WebChem package (version 1.3, Szöcs et al., 2020) and the computing language and environment R (R Core Team, 2020). The compiled LogK_OW_ values are presented in Table S5. To derive the R_Kow_ values, an arithmetic mean of the compound-specific logK_ow_ values was used.

#### In situ sampling rate

Sampling rates vary depending on environmental conditions. Thus, the most accurate estimates of ambient concentrations could be achieved by deriving in situ sampling rates for each sampling site, under relevant conditions (Allinson et al., 2023; Ibrahim et al., 2013; Vrana et al., 2021). Deriving such sampling rates would require frequent grab sampling, increasing the costs drastically.

Here, we estimated in situ R_in situ_ values (Berho et al., 2020) for pharmaceuticals detected in passive samplers and grab water samples at Vanhankaupunginlahti Bay. For each sampler where the compound was detected, we used the mean of concentrations in two grab samples, taken at the start and end of the deployment period of the passive sampler. The R_in situ_ values were estimated using Eq. 3:

3 $${R}_{in situ}=\frac{{C}_{S}{M}_{s}}{{C}_{w}t}$$

where C_S_ is the concentration of pharmaceuticals in the POCIS sampler receiving phase (ng g^−1^), M_S_ is the mass of the sorbent according to the manufacturer (200 mg), C_W_ (ng L^−1^) is the average of concentrations in grab water samples and t is the deployment time in days (d).

## Results and discussion

### Detected substances

The number of analysed substances was between 147 and 150, and altogether, 31 pharmaceuticals were detected from the samples. The number of detected substances was higher in passive samplers (29) than in grab water samples (20). The highest number of pharmaceuticals in passive samplers deployed in the coastal sites was detected at the Vanhankaupunginlahti Bay (24, see Table 1), and the lowest was detected at Eru Bay (8). Fewer compounds were detected in samplers deployed at Estonian coast (14) than the ones at Finnish sites (29). Eight pharmaceuticals were detected at the open sea site (Gulf of Finland), which was assumed to act as a background site with undetectable levels of pharmaceuticals. The detection of pharmaceuticals at the open sea site far from the point pollution sources implies that they are ubiquitous in the environment.

**Fig. 2:**
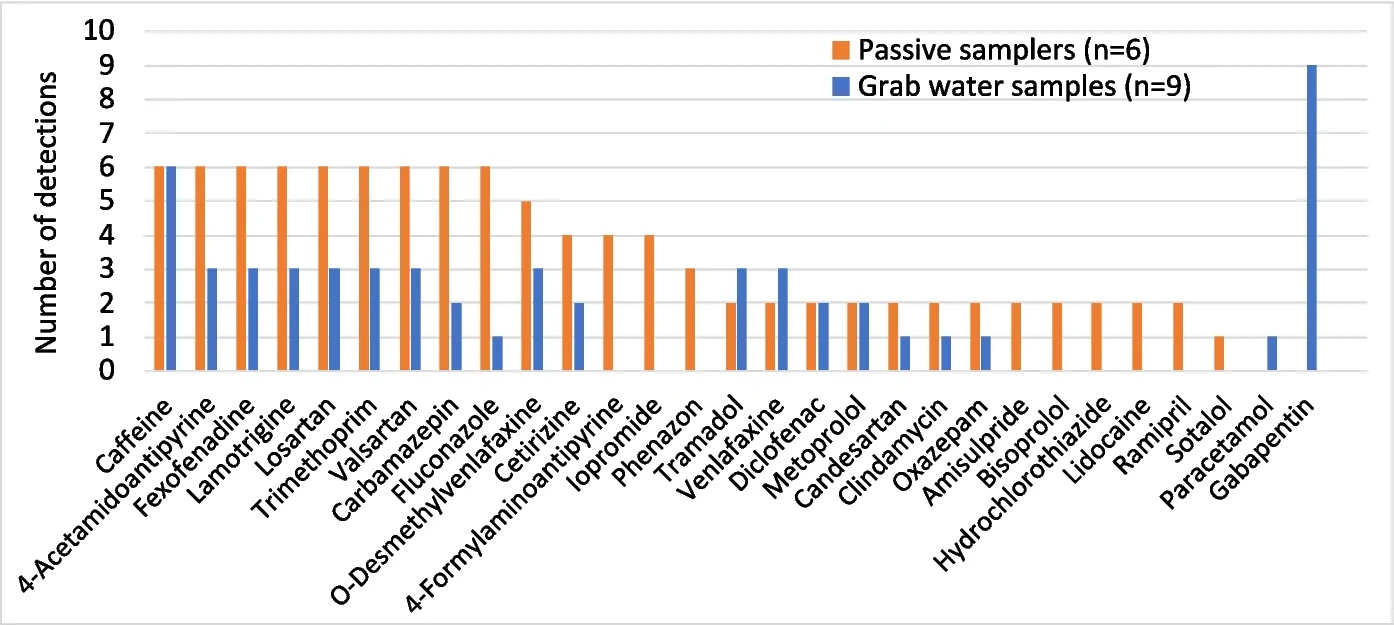
Number of detections of each pharmaceutical in POCIS (*n* = 6) and grab water samples (*n* = 9) taken at the same time at sites at Vanhankaupunginlahti Bay, Kaurakari and Seurasaasenselkä 25.4.−2.7.2024

When comparing the number of pharmaceuticals observed with grab water samples and passive samplers deployed at the same time at Vanhankaupunginlahti Bay, Kaurakari and Seurasaarenselkä, we observed that passive samplers detected 27 compounds and grab water samples detected 20 compounds (Fig. 2). Gabapentin and paracetamol were found only in grab water samples, and gabapentin was discovered in all grab samples. Gabapentin is poorly suitable with HLB material, used in POCIS passive sampler, since it is small and highly polar molecule (Gracia-Lor et al., 2012; Table S5). Additionally, linear accumulation of hydrophilic paracetamol to POCIS HLB sampler has reported to be 3.2 days (Caban et al., 2022). The extraction efficiency from HLB material is low and the mean recovery 14% (Weigel et al., 2004). As a result, POCIS passive sampler with HLB receiving phase is not suitable to study those pharmaceuticals.

### Comparing sampling rates from different sources

**Fig. 3:**
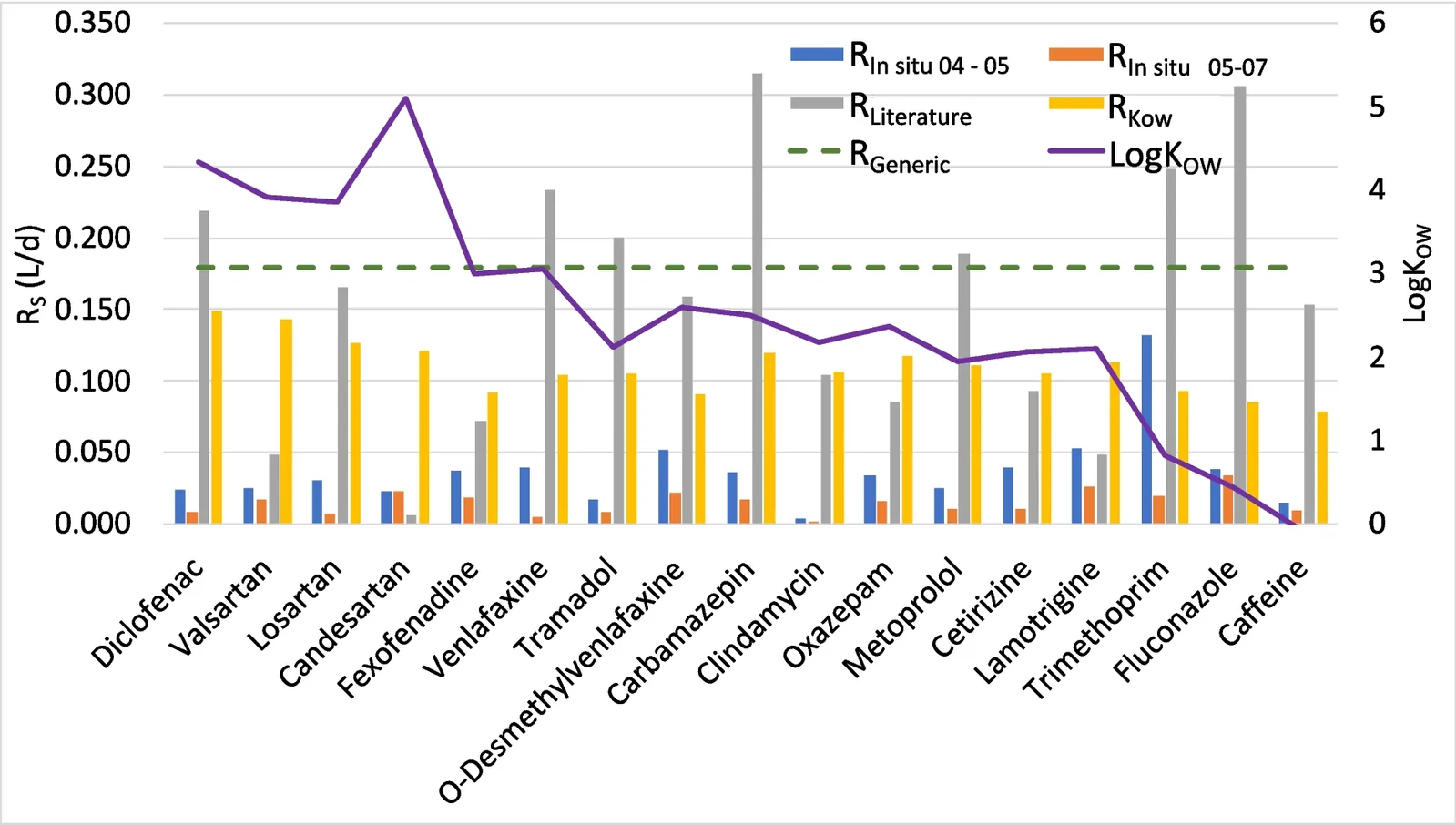
Comparing sampling rates from different sources; R_In situ 04–05_ = in situ sampling rates determined in Vanhankaupunginlahti Bay 25.4.−30.5.2024, R_In situ 05–07_ = in situ sampling rates determined in Vanhankaupunginlahti Bay 30.5.−2.7.2024. R_Literature_ = average of sampling rates collected from the literature, R_Kow_ = sampling rates calculated based on Eq. 2, R_Generic_ = generic sampling rate 0.18 L/d determined by Harman et al. (2012)

Due to consistency, literature sampling rates (R_Literature_) were collected for POCIS passive samplers equipped with HLB receiving phase and deployed in surface water or laboratory conditions (Table S4). Literature sampling rates were not found for all pharmaceuticals detected in this study. The sampling rate for stagnant conditions determined by Harman et al. (2012) was used as a generic sampling rate R_Generic_ = 0.18 L d^−1^. In situ sampling rates R_in situ_ were determined on the Vanhankaupunginlahti Bay sampling site for 17 compounds, which were detected with both passive and grab water sampling (Fig. 3). The difference between the lowest and highest sampling rate reported for the same pharmaceutical ranged from 86 to 99%, which also applies to TWAC differences. Thus, the selection of sampling rates influences on the results.

### Comparing TWACs calculated with different sampling rates

**Fig. 4:**
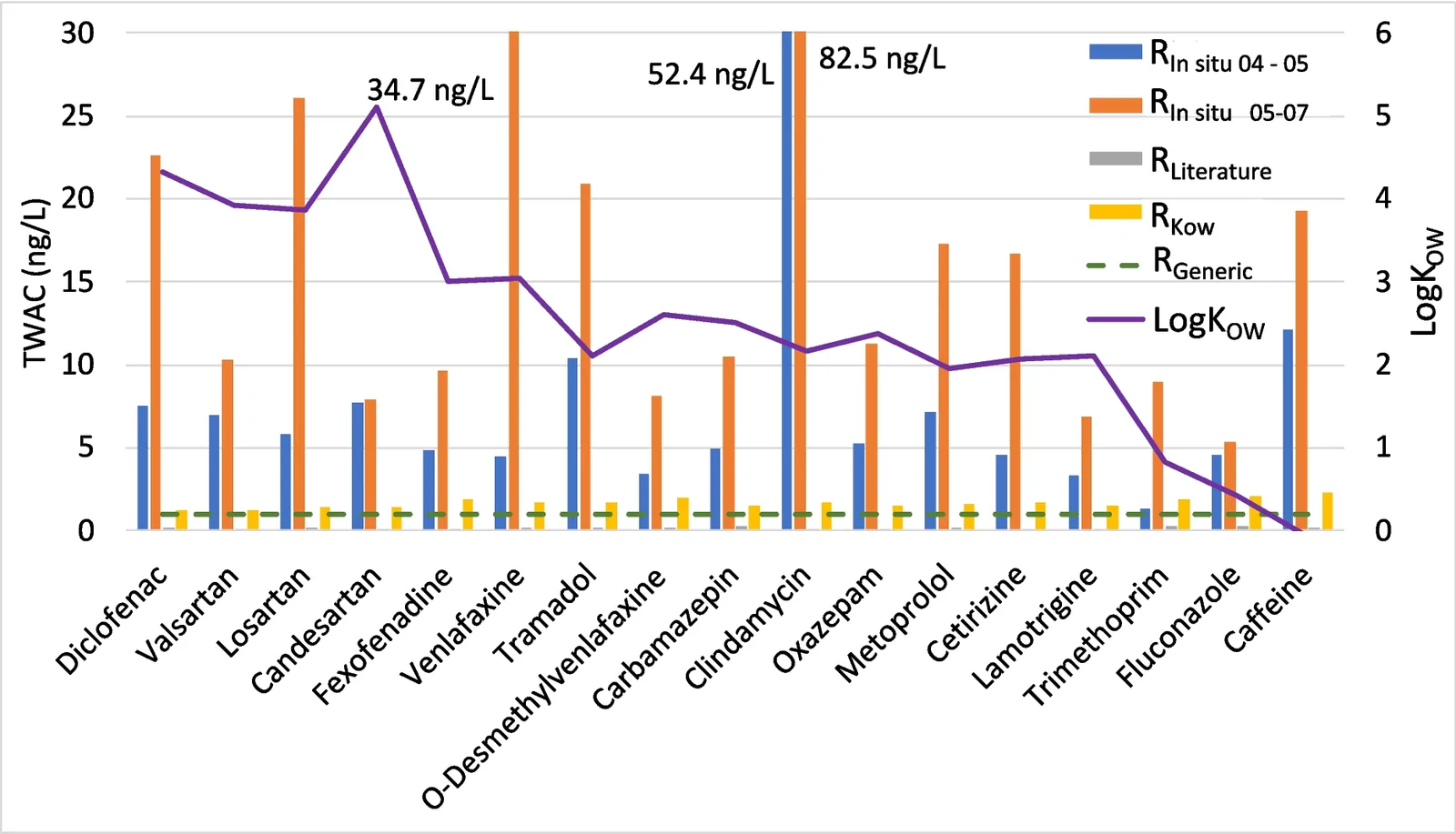
Comparing TWACs calculated for 28 days’ deployment and 5 ng/sampler using different sampling rates: R_In situ 04–05_ = in situ sampling rates determined in Vanhankaupunginlahti Bay 25.4.−30.5.2024, R_In situ 05–07_ = in situ sampling rates determined in Vanhankaupunginlahti Bay 30.5.−2.7.2024. R_Literature_ = average of sampling rates collected from the literature, R_Kow_ = sampling rates calculated based on Eq. 2, R_Generic_ = generic sampling rate 0.18 L/d determined by Harman et al. (2012)

Sampling rates from different sources were utilised to calculate TWACs at same deployment of 28 days and accumulated amount of 5 ng/sampler (Fig. 4). We noticed that R_In situ_ and R_Generic_ yielded higher TWACs than the other ones. LogK_OW_ value of the pharmaceutical did not exert a clear effect on the TWACs which has also been observed by Harman et al., 2012; Li et al., 2010a, 2010b; Wang et al., 2020.

A low concentration of clindamycin was measured in the POCIS samplers, despite high concentration being observed in the grab water samples. This yielded a low in situ sampling rate and a high TWAC (Eq. 1, Fig. 4). However, as R_in situ_ is determined on the basis of long-term passive sampling and instant grab water sampling data, the varying environmental conditions can give diverse sampling rates when compared to sampling rates determined in controlled laboratory conditions. However, concentrations measured from grab water samples can also vary over time being dependent on the sampling occasion.

**Fig. 5:**
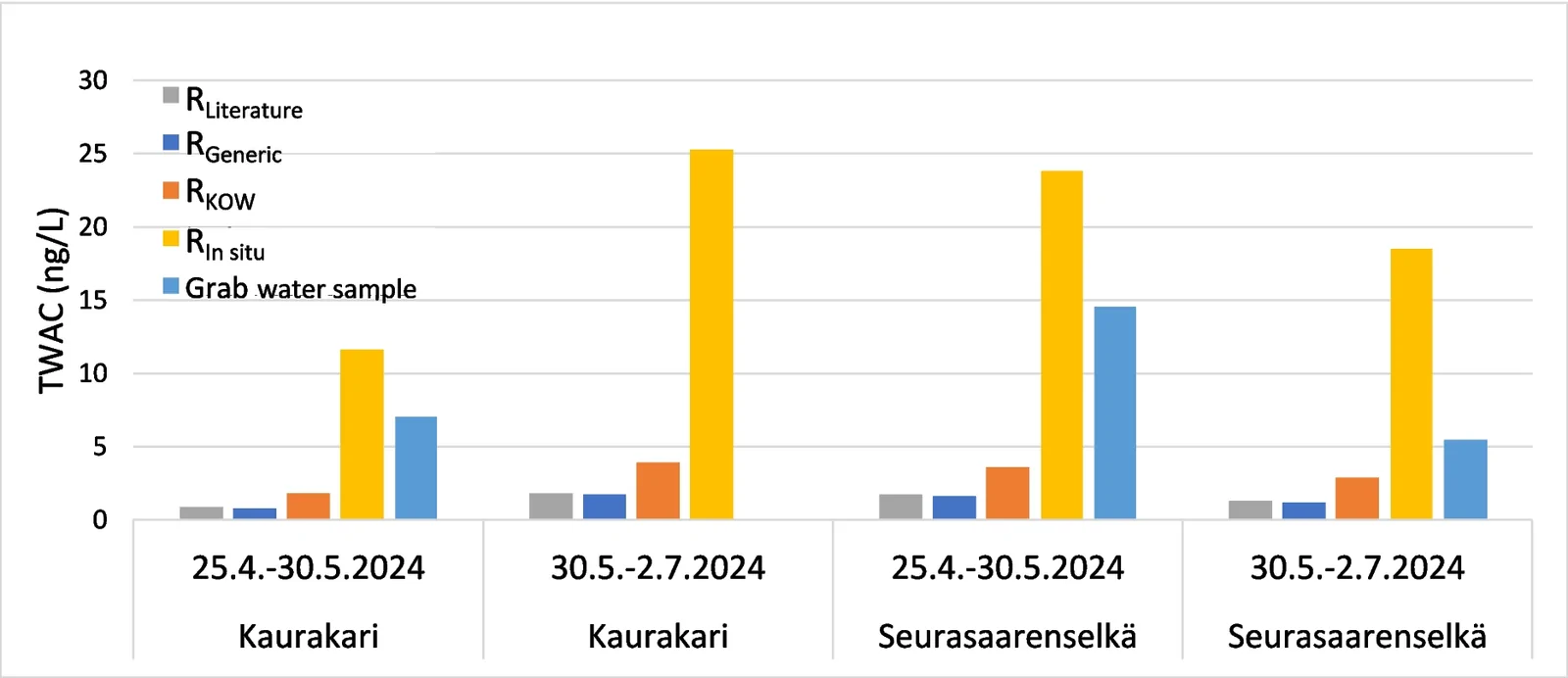
A comparison grab water sample concentrations and the TWACs of caffeine calculated with different sampling rates

To assess the suitability of different sampling rates, TWACs were calculated for caffeine, since it was observed at Seurasaarenselkä and Kaurakari sites, and compared with concentrations measured from grab samples (Fig. 5). R_in situ_ was an average from Vanhankaupunginlahti Bay deployments (Table S3).

The sampling rates collected from the literature or calculated from LogK_OW_ values using Eq. 2 indicated lower TWACs than the concentrations measured in grab water samples (Fig. 5). This is common, as passive samplers collect only the dissolved part of chemical, whereas the total concentration is measured in grab water samples. The sampling rates presented in the literature are usually determined in fixed laboratory conditions (temperature, flow rate) using ultra-pure water. If the field deployment conditions differ from the ones prevailing in laboratory calibration, the sampling rate also differ. R_In_ _situ_ yielded TWACs which were closest to the ones measured in grab water samples (Fig. 5). Caffeine was detected in all passive samplers deployed in this study, but its concentration remained below the LOQ (< 10 ng/L) in three of six grab water samples (Table S2). This suggests that trace concentrations are present in the study area.

In addition to receiving phase materials, field deployment conditions, such as temperature, water turbulence and salinity, exert an effect on the sampling rate of POCIS passive samplers (Harman et al., 2012; Li et al., 2010a, 2010b). Sampling rates of pharmaceuticals and personal care products increase with the flow rate and temperature. However, the effect was generally less than twofold, when the temperature varied between 5–25°C and flow rates were 37 cm s^−1^ and 2.6 cm s^−1^ (Li et al., 2010b) One exception was trimethoprim, for which the sampling rate was 3.44 times higher under higher water flow conditions.

During passive sampler deployments, salinity varied between 5.0 and 5.8‰ at Seurasaarenselkä, 5.4 and 5.7‰ at Kaurakari, 5.6‰ in Espoonlahti Bay and < 0.2 and 2.3 ‰ at Vanhankaupunginlahti Bay (VESLA, 2025). Togola and Budzinski (2007) noted that an increase in salinity decreases the sampling rate of the POCIS sampler. However, the effect of salinity on the sampling rate depends on the chemical character of the compound. For example, the sampling rate of tricyclic antidepressants was reduced by 64% as the salinity increased, while the effect for acidic compounds was not significant. Nevertheless, Vrana et al. (2021) conducted in situ sampling campaigns of pharmaceuticals with varying water flow and temperature conditions and observed no clear changes of sampling rates, but they did not use POCIS with HLB receiving phase.

Additionally, the deployment of samplers in the stainless-steel cage can have an effect on the sampling rate, as the cage can, for instance, slow down the water flow (Lotufo et al., 2018). In laboratory calibrations, the samplers are generally attached parallel to the water flow, and no protective cages are used; thus, the samplers face the water flow straight to the surface of the membrane without any hindrance.

### Concentrations in passive samplers at different sampling sites

**Fig. 6:**
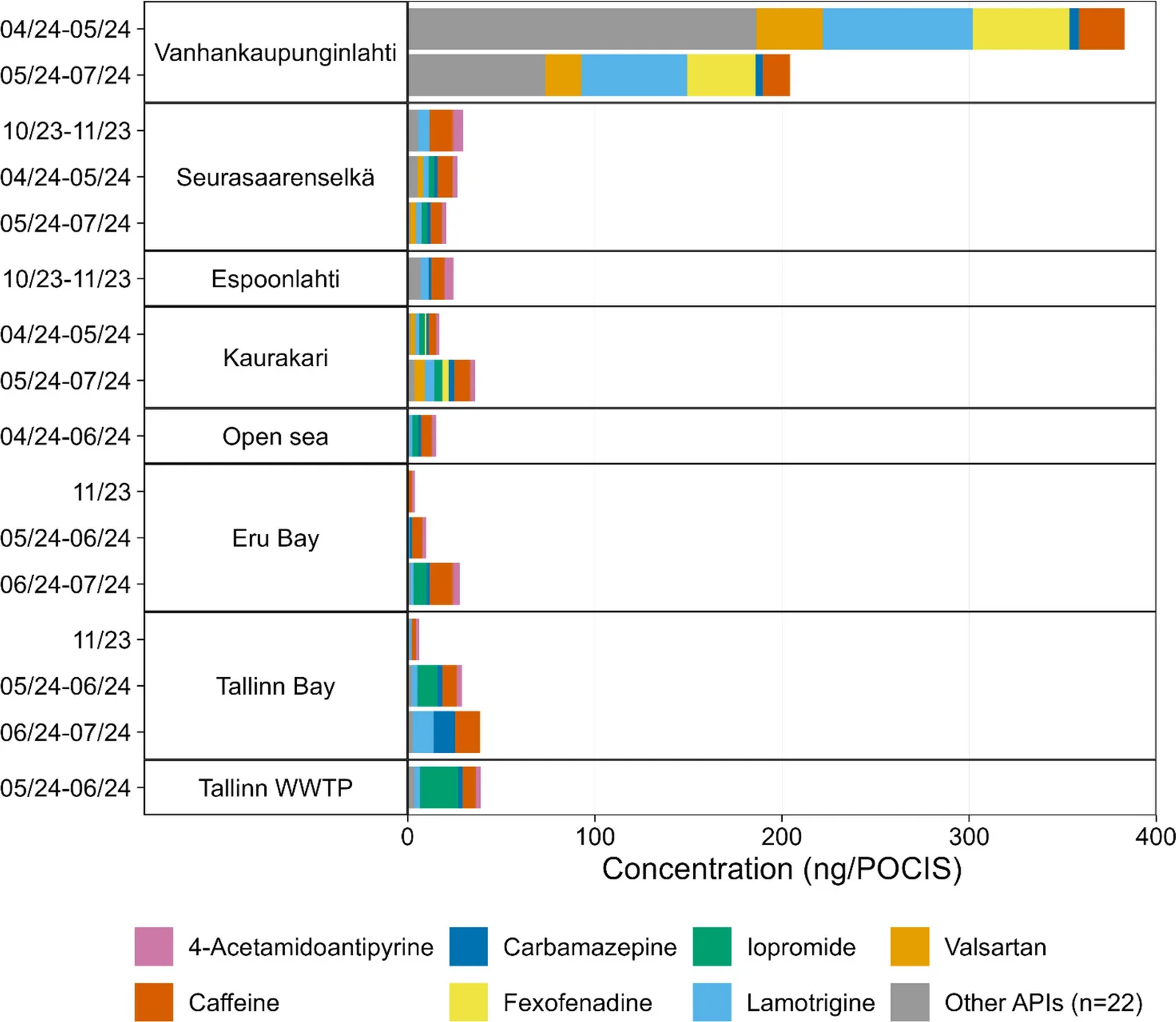
Detected concentrations at different sampling sites for each sampling occasion. The results are calculated for 28-day deployment

**Fig. 7:**
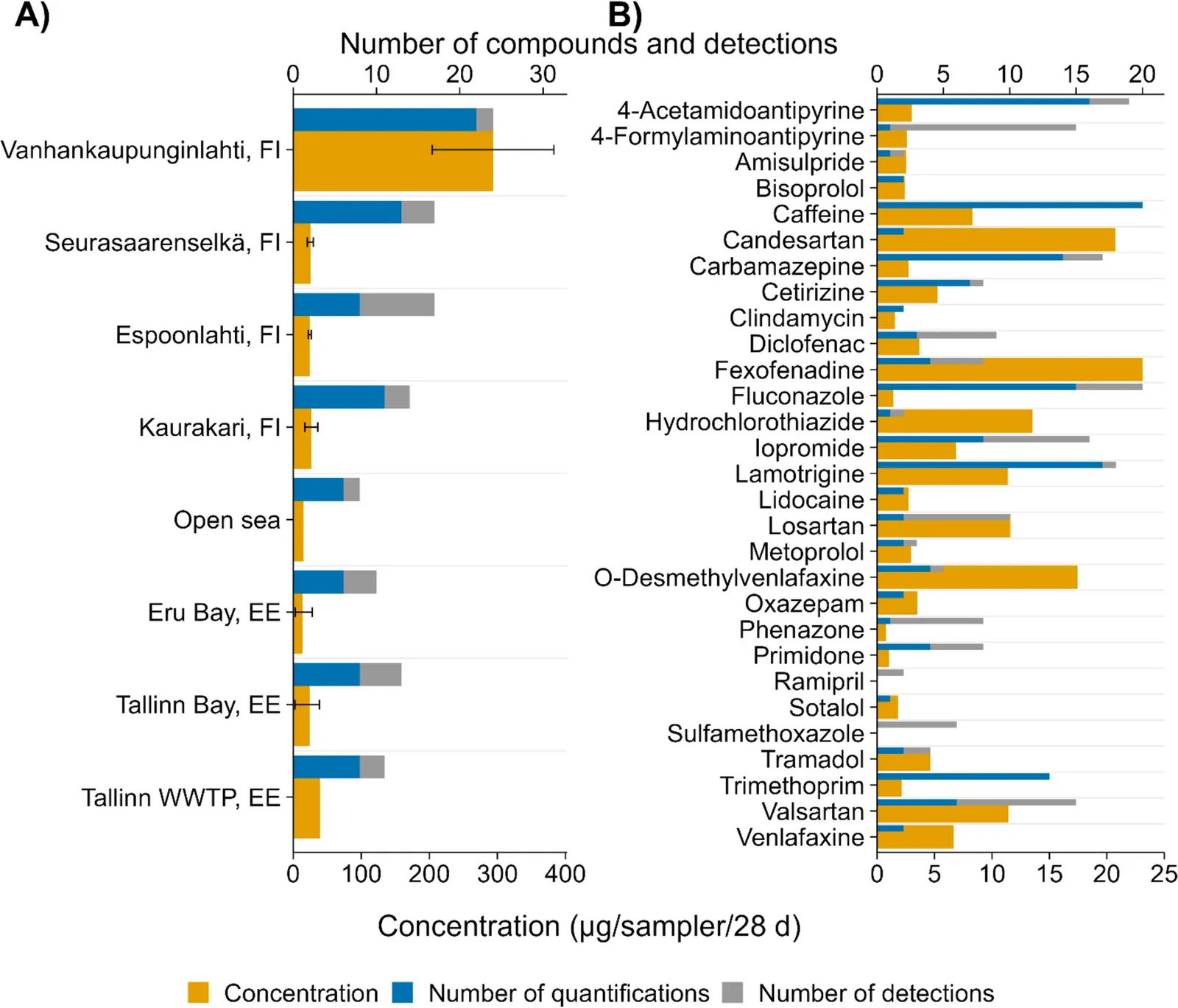
Mean of sum concentrations over sampling events (panel **A**)) and mean concentrations of compounds over all sampling sites (panel **B**)). The results are calculated for 28-day deployment

The concentrations of pharmaceuticals varied between different passive sampling occasions conducted at the same site (Fig. 6, Table S1). For instance, in the Tallinn Bay and Eru Bay, the lowest concentrations were measured in November, but the concentrations were roughly ten times higher in spring and summer. However, at Seurasaarenselkä, the concentrations were roughly at the same level in all sampling occasions, being the highest in November. In November in Espoonlahti Bay, the contents were at about the same level as in Kaurakari. Lamotrigine, caffeine and fexofenadine were detected at the highest concentrations (Fig. 7). In addition, candesartan, cetirizine, losartan and hydrochlorothiazide were detected on many sampling occasions, but their concentrations remained low. Iopromide, which is used in x-ray contrast agents, was detected at the Tallinn WWTP, Tallinn Bay and Eru Bay sites. This implies releases from several hospitals located at Tallinn area.

The mean of sum concentrations over each sampling site were the highest at Vanhankaupunginlahti Bay, which receives waters from River Vantaanjoki, which is rather polluted (Fig. 7). Site Tallinn WWTP was expected to have a high concentration of pharmaceuticals, but the results reveal that the concentrations were about the same level as at other sites, excluding Vanhankaupunginlahti Bay. Eru Bay was considered as a background site based on lack of pollution sources. The results confirmed this, as the lowest concentrations were measured in Eru Bay, and the number of detected substances was among the lowest.

During the second deployment at the Vanhankaupunginlahti Bay (30.5.−2.7.2024), a high a degree of biofouling developed on the deployment cage, which most likely reduced the accumulation of pharmaceuticals in the passive samplers.

**Fig. 8:**
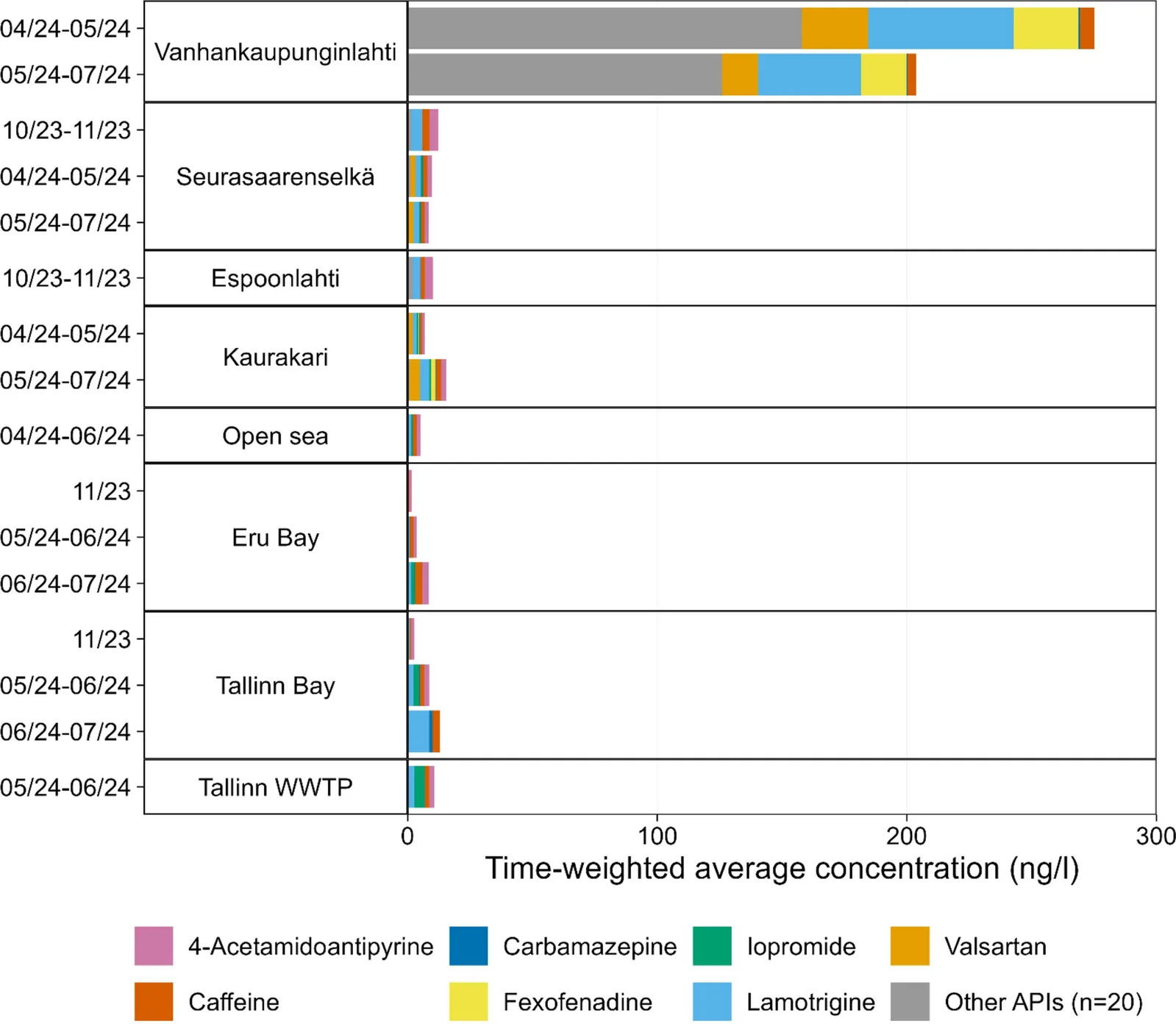
TWACs at different sampling sites for each sampling occasion calculated using the generic sampling rate R_Generic_ = 0.18 L d^−1^

As TWACs (Fig. 8) were calculated using the generic sampling rate R_Generic_ = 0.18 L d^−1^, and the ratio of the pharmaceuticals remained the same as in Fig. 6. In the Vanhankaupunginlahti Bay, the sum concentration of pharmaceuticals varied between about 200 and 270 ng L^−1^, but at other sites, the sum remained below 10 ng L^−1^.

### Preliminary risk assessment

The toxic effect of a chemical is assessed by utilising the chemical-specific no-effect concentration (PNEC), which is considered as a limit below which no adverse effects occur in organisms. PNEC is derived based on laboratory assays of different trophic levels which were algae, crustaceans or fish. The risk that chemicals pose to the aquatic environment is commonly evaluated based on the risk quotient (RQ), for which the measured environmental concentration is divided by the PNEC of a studied chemical (Finckh et al., 2022; Zhang et al., 2016). The availability and amount of toxicity test data varies, bringing greater uncertainty to toxicity estimations. From an ecotoxicological perspective, passive samplers mimic the uptake of chemicals to organisms, and the estrogen activity of POCIS extract and bile of fish has been observed to be correlated (Harman et al., 2012; Vermeirssen et al., 2005) Silicone rubber passive samplers can also predict conservative estimations of hydrophobic compounds in fish (Allan et al., 2025).

TWACs (Table S3) were divided with PNEC values (Table S6) to determine RQ. According to the FASS (2022) database, the 0.1 < RQ < 1 indicates low environmental risk, and according to EMA (2006), guideline RQ > 1 requires further ecotoxicological studies of pharmaceuticals. Alvarez et al. (2021) recommended conservative approach of RQ > 0.1 to consider the full variability of the measured concentrations. TWAC based RQs above 0.1 were obtained for carbamazepine (0.16–0.46) and diclofenac (0.41), using TWAC calculated with R_in situ_, which presents a low or insignificant risk. For other pharmaceuticals the TWAC based RQs remained below 0.1.

When assessing the possible risk based on grab sampling results, the RQs were higher than ones based on TWACs (Table S2 and S6). This may be due to higher detection limits in grab water samples and the selection of sampling rates used in TWAC calculations. The highest RQ of 0.3 was observed for diclofenac, and RQ of 0.27 was observed for venlafaxine, both indicating low environmental risk.

### Passive sampling in regulatory monitoring

Interest in the application of passive sampling in regulatory monitoring has increased (Allan et al., 2025). There are open questions about how to reconcile the passive sampling results with regulatory monitoring obligations. The grab and passive sampling techniques are different, as one is instant and the other is long term, one provides total concentrations of chemicals, and the other the dissolved part, which is the most deleterious to aquatic organisms. Reconciling these two sampling techniques is a challenge. The threshold concentrations, such as PNECs or the EU Water Framework Directive’s Environmental Quality Standard values (WFD EQS, European Commission, 2000; EU Parliament Council, 2008) are typically total concentrations determined from instant grab water samples. To use passive sampling as a reliable tool in regulatory monitoring, the determination of reference concentrations with passive samplers should be discussed. One option to overcome this could be that with passive sampling, the differences between the concentrations determined at sampling sites are studied.

One particularly useful application of passive sampling technique is the deployment of samplers both upstream and downstream of a polluter´s discharge point, cost efficiently revealing the contaminants released into the water (Ahkola et al., 2022; Lentola et al., 2025). Additionally, an estimation of a baseline concentration determined from samplers deployed at a background site would be beneficial. Baseline concentrations could be determined for different areas, and the results from suspected polluters or hot spots could be compared with those used to recognise the possible environmental risk. In the presence of diffuse pollution, baseline concentrations may contain studied compounds- However the difference in concentration levels compared to possible hotspot sites is essential.

The passive sampling could also be utilized in selection of monitoring points and pharmaceuticals to be further monitored in order to wisely focus the limited monitoring resources; however, before that, the linkage to regulatory limit values should be settled.

The calibration of different passive sampling techniques should be further developed to enable more convenient assessment of TWACs. Although existing studies attempted to harmonise TWACs with grab water sampling concentrations, this may not be necessary. Instead, the issue could be reconsidered by avoiding attempts to force comparability between methods and by re-evaluating the basis of regulatory limit values. EQS values could be compared conveniently with TWACs, as both consider a non-existent or negligible amount of particle-bound chemical fraction.

## Conclusions

The passive sampling technique enriched the trace concentrations of some pharmaceuticals to measurable levels, allowing the detection of more pharmaceuticals compared to traditional grab water sampling- The highest number of pharmaceuticals in passive samplers deployed in the coastal sites was detected at the Vanhankaupunginlahti Bay off the coast of Helsinki. Eight pharmaceuticals were detected at the open sea area of Gulf of Finland, considered as a background site. Thus, determining baseline concentrations for the regulatory monitoring of pharmaceuticals should be further developed.

TWAC determined with in situ sampling rates from sites Vanhankaupunginlahti Bay, yielded concentrations closest to the ones determined in grab water samples at Kaurakari and Seurasaari sites. This suggests that despite the different locations of the sampling sites, their environmental conditions did not differ significantly. Therefore, the findings of this study support the use of in situ sampling rate.

The deployment time of passive samplers should be carefully evaluated as at some sites substantial biofouling in sampling cages and passive samplers may occur. At those sites, the deployment time should not exceed a three-weeks, at least in summertime.

The risk assessment indicated only an insignificant risk for carbamazepine and diclofenac, but as TWACs represent dissolved fraction and the thresholds are expressed as total concentration, the comparison is not necessarily rational. There are open questions about how to reconcile the passive sampling results with regulatory monitoring obligations and existing threshold values, such as WFD EQS values and PNECs. To use passive sampling as a reliable tool in environmental monitoring, the basis of reference concentrations should be re-considered to be more representative in relation to passive sampling results.

Both, passive and grab sampling methods have their pros and cons. There are still open questions about how to reconcile the passive sampling results with regulatory monitoring obligations based on grab water sampling and existing limit concentrations. Long-term average concentration estimates produced by passive sampling may not capture the complete picture of contaminant presence in the environment; however, in environmental research, a certain degree of uncertainty must be accepted (Ahkola et al., 2024). The key question is what kind of uncertainty is considered the most acceptable.

## Supplementary Information

Below is the link to the electronic supplementary material.

ESM 1 (XLSX 441 KB)

ESM 2 (DOCX 72.0 KB)

## Data Availability

Research data is presented as supplementary material.

## References

[CR1] Ahkola, H., Juntunen, J., Krogerus, K., & Huttula, T. (2022). Monitoring and modelling of butyltin compounds in Finnish inland lake. *Frontiers in Environmental Chemistry*. 10.3389/fenvc.2022.1063667

[CR2] Ahkola, H., Kotamäki, N., Siivola, E., Tiira, J., Imoscopi, S., Riva, M., Tezel, U., & Juntunen, J. (2024). Uncertainty in environmental micropollutant modeling. *Environmental Management,**74*(2), 380–398. 10.1007/s00267-024-01989-z 38816505 PMC11227446

[CR3] Allan, I. J., Miege, C., Jahnke, A., Rojo-Nieto, E., Vorkamp, K., Kech, C., Polesello, S., Perceval, O., Booij, K., Dulio, V., Estoppey, N., Mayer, P., Mchugh, B., Munschy, C., Staub, P. F., & Vrana, B. (2025). Passive sampling in support of biota monitoring of hydrophobic substances under the water framework directive. *Journal of Hazardous Materials*. 10.1016/j.jhazmat.2024.136672 39615388

[CR4] Allinson, M., Cassidy, M., Kadokami, K., & Besley, C. H. (2023). In situ calibration of passive sampling methods for urban micropollutants using targeted multiresidue GC and LC screening systems. *Chemosphere,**311*(Pt 1), Article 136997. 10.1016/j.chemosphere.2022.136997 36309053

[CR5] Alvarez, D. A., Corsi, S. R., Cicco, D., Laura, A., Villeneuve, D. L., & Baldwin, A. K. (2021). Identifying chemicals and mixtures of potential biological concern detected in passive samplers from Great Lakes tributaries using high-throughput data and biological pathways. *Environmental Toxicology and Chemistry,**40*(8), 2165–2182. 10.1002/etc.5118 34003517 PMC8361951

[CR6] Alvarez, D. A., Petty, J. D., Huckins, J. N., Jones-Lepp, T. L., Getting, D. T., Goddard, J. P., & Manahan, S. E. (2004). Development of a passive, in situ, integrative sampler for hydrophilic organic contaminants in aquatic environments. *Environmental Toxicology and Chemistry,**23*(7), 1640–1648. 10.1897/03-603 15230316

[CR7] Baumgartner, S., Salvisberg, M., Clot, B., Crouzy, B., Schmid-Grendelmeier, P., Singer, H., & Ort, C. (2024). *Wastewater-based analysis of antihistamines to investigate pollinosis symptom burden at population-scale*. medRxiv,2023.11.29.23299171.

[CR8] Bayen, S., Segovia, E., Loh, L. L., Burger, D. F., Eikaas, H. S., & Kelly, B. C. (2014). Application of polar organic chemical integrative sampler (POCIS) to monitor emerging contaminants in tropical waters. *Science of the Total Environment, 482*, 15–22. 10.1016/j.scitotenv.2014.02.082 24632061

[CR9] Berho, C., Robert, S., Coureau, C., Coisy, E., Berrehouc, A., Amalric, L., & Bruchet, A. (2020). Estimating 42 pesticide sampling rates by POCIS and POCIS-MIP samplers for groundwater monitoring: A pilot-scale calibration. *Environmental Science and Pollution Research,**27*(15), 18565–18576. 10.1007/s11356-020-08385-0 32198689

[CR10] Booij, K., & Chen, S. (2018). Review of atrazine sampling by polar organic chemical integrative samplers and Chemcatcher. *Environmental Toxicology and Chemistry,**37*(7), 1786–1798. 10.1002/etc.4160 29687480

[CR11] Caban, M., Lis, H., & Stepnowski, P. (2022). Limitations of integrative passive samplers as a tool for the quantification of pharmaceuticals in the environment - A critical review with the latest innovations. *Critical Reviews in Analytical Chemistry,**52*(6), 1386–1407. 10.1080/10408347.2021.1881755 33673780

[CR12] Chen, Y., Huang, Y., Chen, Y., Li, Y., Sidikjan, N., Lin, N., Li, Y., Guo, X., Shen, G., & Liu, M. (2025). A systematic review of sources, occurrence, behavior and risks of global marine antibiotics. *Npj Emerging Contaminants,**1*(1), Article 15. 10.1038/s44454-025-00015-z

[CR13] Domínguez-García, P., Almirall, X. O., & Gómez-Canela, C. (2025). A POCIS-based approach for the monitoring of pharmaceuticals in wastewater treatment plants: Calibration and deployment challenges. *Environmental Pollution,**367*, Article 125641. 10.1016/j.envpol.2025.125641 39798795

[CR14] EMA. (2006). *European medicines agency: Guideline on the environmental risk assessment of medicinal products for human use* (p. 12). Committee for Medicinal Products for Human Use (CHMP). https://www.ema.europa.eu/en/documents/scientific-guideline/guideline-environmental-risk-assessment-medicinal-products-human-use-revision-1_en.pdf

[CR15] EU Parliament Council. (2008). Directive 2008/105/EC of the European Parliament and of the Council of 16December 2008 on environmental quality standards in the field of water policy, amending and subsequently repealing Council Directives 82/176/EEC, 83/513/EEC, 84/156/ EEC, 84/491/EEC, 86/280/EEC and amending Directive 2000/60/EC of the European Parliament and of the Council. *Official Journal of European Union, L348*, 84–97.

[CR16] European Commission. (2000). *Directive 2000/60/EC of the European parliament and of the council of 23 October2000 Establishing a framework for community action in the field of water policy*. Official Journal 22 December 2000L 327/1. European Commission.

[CR17] Fass. (2022). Fass website, environmental risk documents (via Felleskatalogen) https://www.felleskatalogen.no/medisin/miljo/legemiddel/alle (Accessed 29.12.2022).

[CR18] Finckh, S., Beckers, L. M., Busch, W., Carmona, E., Dulio, V., Kramer, L., Krauss, M., Posthuma, L., Schulze, T., Slootweg, J., von der Ohe, P. C., & Brack, W. (2022). A risk based assessment approach for chemical mixtures from wastewater treatment plant effluents. *Environment International*. 10.1016/j.envint.2022.107234 35483182

[CR19] Gracia-Lor, E., Martínez, M., Sancho, J. V., Peñuela, G., & Hernández, F. (2012). Multi-class determination of personal care products and pharmaceuticals in environmental and wastewater samples by ultra-high performance liquid-chromatography-tandem mass spectrometry. *Talanta,**99*, 1011–1023. 10.1016/j.talanta.2012.07.091 22967656

[CR20] Harman, C., Allan, I. J., & Vermeirssen, E. L. M. (2012). Calibration and use of the polar organic chemical integrative sampler-a critical review. *Environmental Toxicology and Chemistry,**31*(12), 2724–2738. 10.1002/etc.2011 23012256

[CR21] Harman, C., Reid, M., & Thomas, K. V. (2011). In situ calibration of a passive sampling device for selected illicit drugs and their metabolites in wastewater, and subsequent year-long assessment of community drug usage. *Environmental Science & Technology,**45*(13), 5676–5682. 10.1021/es201124j 21648435

[CR22] Ibrahim, I., Togola, A., & Gonzalez, C. (2013). In-situ calibration of POCIS for the sampling of polar pesticides and metabolites in surface water. *Talanta,**116*, 495–500. 10.1016/j.talanta.2013.07.028 24148435

[CR23] Kim, S., Chen, J., Cheng, T., Gindulyte, A., He, J., He, S., Li, Q., Shoemaker, B. A., Thiessen, P. A., Yu, B., Zaslavsky, L., Zhang, J., & Bolton, E. E. (2025). PubChem 2025 update. *Nucleic Acids Research,**53*(D1), D1516–D1525. 10.1093/nar/gkae1059 39558165 PMC11701573

[CR24] Lentola, A., Lunger, A., Rottensteiner, A., Spitaler, R., & Bonadio, M. (2025). Passive samplers in surface water: A case-based evaluation of their use for point source pollution detection. *Environmental Sciences Europe*. 10.1186/s12302-025-01191-w

[CR25] Leppäranta, M., & Myrberg, K. (2009). Physical oceanography of the Baltic Sea. *Physical Oceanography of the Baltic Sea*, 1–378. 10.1007/978-3-540-79703-6

[CR26] Li, H. X., Helm, P. A., & Metcalfe, C. D. (2010b). Sampling in the Great Lakes for pharmaceuticals, personal care products, and endocrine-disrupting substances using the passive polar organic chemical integrative sampler. *Environmental Toxicology and Chemistry,**29*(4), 751–762. 10.1002/etc.104 20821503

[CR27] Li, H., Vermeirssen, E. L., Helm, P. A., & Metcalfe, C. D. (2010a). Controlled field evaluation of water flow rate effects on sampling polar organic compounds using polar organic chemical integrative samplers. *Environmental Toxicology and Chemistry,**29*(11), 2461–2469. 10.1002/etc.305 20865700

[CR28] Lindholm-Lehto, P. C., Ahkola, H. S. J., Knuutinen, J. S., & Herve, S. H. (2016). Widespread occurrence and seasonal variation of pharmaceuticals in surface waters and municipal wastewater treatment plants in central Finland. *Environmental Science and Pollution Research,**23*(8), 7985–7997. 10.1007/s11356-015-5997-y 26769590

[CR29] Lotufo, G. R., George, R. D., Belden, J. B., Woodley, C. M., Smith, D. L., & Rosen, G. (2018). Investigation of polar organic chemical integrative sampler (POCIS) flow rate dependence for munition constituents in underwater environments. *Environmental Monitoring and Assessment*. 10.1007/s10661-018-6558-x 29478103

[CR30] Martínez-Megías, C., Arenas-Sánchez, A., Manjarrés-López, D., Pérez, S., Soriano, Y., Picó, Y., & Rico, A. (2024). Pharmaceutical and pesticide mixtures in a Mediterranean coastal wetland: Comparison of sampling methods, ecological risks, and removal by a constructed wetland. *Environmental Science and Pollution Research*. 10.1007/s11356-024-31968-0 PMC10884053 38277107

[CR31] Myrberg, K., & Soomere, T. (2013). The Gulf of Finland, its hydrography and circulation dynamics. In T. Soomere & E. Quack (Eds.), *Preventive methods for coastal protection: Towards the use of ocean dynamics for pollution control* (pp. 181–222). Springer-Verlag. 10.1007/978-3-319-00440-2_6

[CR32] Poulier, G., Lissalde, S., Charriau, A., Buzier, R., Cleries, K., Delmas, F., Mazzella, N., & Guibaud, G. (2015). Estimates of pesticide concentrations and fluxes in two rivers of an extensive French multi-agricultural watershed: Application of the passive sampling strategy. *Environmental Science and Pollution Research,**22*(11), 8044–8057. 10.1007/s11356-014-2814-y 24777319

[CR33] R Core Team. (2020). R: A language and environment for statistical computing. R Foundation for Statistical Computing, Vienna, Austria. https://www.R-project.org/.

[CR34] Rico, A., Arenas-Sánchez, A., Alonso-Alonso, C., López-Heras, I., Nozal, L., Rivas-Tabares, D., & Vighi, M. (2019). Identification of contaminants of concern in the upper Tagus river basin (central Spain). Part 1: Screening, quantitative analysis and comparison of sampling methods. *Science of the Total Environment,**666*, 1058–1070. 10.1016/j.scitotenv.2019.02.250 30970472

[CR35] Röemerscheid, M., Paschke, A., Schneider, S., Blaha, M., Harzdorf, J., & Schüeüermann, G. (2023). Calibration of the Chemcatcher? passive sampler and derivation of generic sampling rates for a broad application in monitoring of surface waters. *Science of the Total Environment, 871*. 10.1016/j.scitotenv.2023.161936 36746283

[CR36] Szöcs, E., Stirling, T., Scott, E. R., Scharmüller, A., & Schäfer, R. B. (2020). Webchem: An r package to retrieve chemical information from the web. *Journal of Statistical Software,**93*(13), 1–17. 10.18637/jss.v093.i13

[CR37] Taylor, A. C., Mills, G. A., Gravell, A., Kerwick, M., & Fones, G. R. (2021). Passive sampling with suspect screening of polar pesticides and multivariate analysis in river catchments: Informing environmental risk assessments and designing future monitoring programmes. *Science of the Total Environment*. 10.1016/j.scitotenv.2021.147519 33992941

[CR38] Togola, A., & Budzinski, H. (2007). Development of polar organic integrative samplers for analysis of pharmaceuticals in aquatic systems. *Analytical Chemistry,**79*(17), 6734–6741. 10.1021/ac070559i 17683162

[CR39] Tousová, Z., Vrana, B., Smutná, M., Novák, J., Klucárová, V., Grabic, R., Slobodník, J., Giesy, J. P., & Hilscherová, K. (2019). Analytical and bioanalytical assessments of organic micropollutants in the Bosna River using a combination of passive sampling, bioassays and multi-residue analysis. *Science of the Total Environment,**650*, 1599–1612. 10.1016/j.scitotenv.2018.08.336 30308846

[CR40] USEPA. (2007). *Method 1694: Pharmaceuticals and personal care products in water, soil, sediment, and biosolids by HPLC/MS/MS*. USEPA. https://www.epa.gov/sites/production/files/2015-10/documents/method_1694_2007.pdf. Accessed 3.8.2026.

[CR41] Vermeirssen, E. L. M., Körner, O., Schönenberger, R., Suter, M. J. F., & Burkhardt-Holm, P. (2005). Characterization of environmental estrogens in river water using a three pronged approach: Active and passive water sampling and the analysis of accumulated estrogens in the bile of caged fish. *Environmental Science & Technology,**39*(21), 8191–8198. 10.1021/es050818q 16294854

[CR42] VESLA. (2025). Water quality of surface waters -Register. *Accessed,**15*(1), 2025.

[CR43] Vieno, N. M., Tuhkanen, T., & Kronberg, L. (2005). Seasonal variation in the occurrence of pharmaceuticals in effluents from a sewage treatment plant and in the recipient water. *Environmental Science & Technology,**39*(21), 8220–8226. 10.1021/es051124k 16294858

[CR44] Vrana, B., Urík, J., Fedorova, G., Svecová, H., Grabicová, K., Golovko, O., Randák, T., & Grabic, R. (2021). In situ calibration of polar organic chemical integrative sampler (POCIS) for monitoring of pharmaceuticals in surface waters. *Environmental Pollution, 269*. 10.1016/j.envpol.2020.116121 33272798

[CR45] Wang, L. Y., Liu, R. X., Liu, X. L., & Gao, H. J. (2020). Sampling rate of polar organic chemical integrative sampler (POCIS): Influence factors and calibration methods. *Applied Sciences*. 10.3390/app10165548

[CR46] Weigel, S., Kallenborn, R., & Hühnerfuss, H. (2004). Simultaneous solid-phase extraction of acidic, neutral and basic pharmaceuticals from aqueous samples at ambient (neutral) pH and their determination by gas chromatography-mass spectrometry. *Journal of Chromatography A,**1023*(2), 183–195. 10.1016/j.chroma.2003.10.036 14753684

[CR47] Zandaryaa, S., & Frank-Kamenetsky, D. (2021). A source-to-sea approach to emerging pollutants in freshwater and oceans: Pharmaceuticals in the Baltic Sea region. *Source-to-sea management* (pp. 60–75). Routledge.

[CR48] Zhang, Z. L., Troldborg, M., Yates, K., Osprey, M., Kerr, C., Hallett, P. D., Baggaley, N., Rhind, S. M., Dawson, J. J. C., & Hough, R. L. (2016). Evaluation of spot and passive sampling for monitoring, flux estimation and risk assessment of pesticides within the constraints of a typical regulatory monitoring scheme. *Science of the Total Environment,**569*, 1369–1379. 10.1016/j.scitotenv.2016.06.219 27425435

