# Supplementary material for: Marine pharmaceutical monitoring with POCIS: sampling rate uncertainty, and implications for regulation
Source: Environ Monit Assess. 2026 Aug 8;198(9):926. doi: 10.1007/s10661-026-15742-y (PMC13452753; doi:10.1007/s10661-026-15742-y)
Supplement: Supplementary file 1 [file 10661_2026_15742_MOESM2_ESM.docx]

# Supplementary information

Table S4. Sampling rates R_Literature_ (L/day) collected from literature for pharmaceuticals detected from POCIS passive samplers with HLB receiving phase and in surface water or laboratory conditions.

| **Substance** | **Elkayar et al. 2022** | **Li et al. 2010** | **Li et al. 2010** | **Harman et al. 2011** | **Togola and Budzinski, 2007** | **Morin et al. 2013** | **MacLeod et al. 2007** | **Guibal et al. 2020** | **Guibal et al. 2020** | **Bartelt-Hunt et al. 2011** | **Bailly et al. 2013** | **Bailly et al. 2013** | **Morin et al. 2012** | **Bayen et al. 2014** | **Magi et al. 2018** | **Wang et al. 2022** | **Criquet et al. 2017** | **Allinson et al. 2023** | **Allinson et al. 2023** | **Vrana et al. 2021** | **Domínguez-García et al. 2025** | **Average** | **Standard deviation** |
| --- | --- | --- | --- | --- | --- | --- | --- | --- | --- | --- | --- | --- | --- | --- | --- | --- | --- | --- | --- | --- | --- | --- | --- |
| 4-Acetamidoantipyrine |  |  |  |  |  |  |  |  |  |  |  |  |  |  |  |  |  |  |  |  | 0.053 | 0.053 |  |
| 4-Formylaminoantipyrine |  |  |  |  |  |  |  |  |  |  |  |  |  |  |  |  |  |  |  |  |  |  |  |
| Amisulpride |  |  |  |  |  |  |  |  |  |  |  |  |  |  |  |  |  |  |  |  |  |  |  |
| Benzotriazole |  |  |  |  |  |  |  |  |  |  |  |  |  |  |  | 0.043 |  |  |  |  |  | 0.043 |  |
| Bisoprolol |  |  |  |  |  |  |  |  | 0.200 |  |  |  |  |  |  |  |  |  |  |  |  | 0.200 |  |
| Diclofenac |  |  |  | 0.166 | 0.275 |  |  | 0.334 | 0.239 |  |  |  |  |  |  |  | 0.080 |  |  |  |  | 0.219 | 0.099 |
| Fexofenadine |  |  |  |  |  |  |  |  |  |  |  |  |  |  |  | 0.091 |  |  |  | 0.052 |  | 0.072 | 0.028 |
| Phenazon |  |  |  |  |  |  |  |  |  |  |  |  |  |  |  |  |  |  |  |  |  |  |  |
| Fluconazole | 0.306 |  |  |  |  |  |  |  |  |  |  |  |  |  |  |  |  |  |  |  |  | 0.306 |  |
| Hydrochlorothiazide |  |  |  |  |  |  | 0.053 |  |  |  |  |  |  |  |  |  |  |  |  |  |  | 0.053 |  |
| Candesartan |  |  |  |  |  |  |  |  |  |  |  |  |  |  |  |  |  | 0.011 | 0.002 |  |  | 0.007 | 0.006 |
| Carbamazepin | 0.214 | 0.397 | 0.230 |  |  | 0.188 | 0.348 | 0.443 | 0.299 | 0.288 |  |  |  | 0.600 |  | 0.141 |  |  |  |  |  | 0.315 | 0.138 |
| Clindamycin |  |  |  |  |  |  |  |  |  |  |  |  |  |  |  |  |  |  |  | 0.104 |  | 0.104 |  |
| Caffeine | 0.170 | 0.151 | 0.096 |  |  |  |  |  |  | 0.044 |  |  |  | 0.550 | 0.141 | 0.016 |  |  |  |  | 0.055 | 0.153 | 0.170 |
| Lamotrigine |  |  |  |  |  |  |  |  |  |  |  |  |  |  |  | 0.049 |  |  |  |  |  | 0.049 |  |
| Lidocaine |  |  |  |  |  |  |  |  |  |  |  |  |  |  |  | 0.120 |  |  |  |  | 0.189 | 0.155 | 0.049 |
| Losartan |  |  |  |  |  |  |  |  |  |  |  |  |  |  |  | 0.041 |  |  |  |  | 0.291 | 0.166 | 0.177 |
| Metoprolol |  | 0.309 |  |  |  |  |  |  |  |  |  |  |  |  |  | 0.070 |  |  |  |  |  | 0.190 | 0.169 |
| Oxazepam |  |  |  |  |  |  |  |  |  |  |  |  |  |  |  |  |  |  |  | 0.085 |  | 0.085 |  |
| Primidone |  |  |  |  |  |  |  |  |  |  |  |  |  |  |  |  |  |  |  |  |  |  |  |
| Ramipril |  |  |  |  |  |  |  |  |  |  |  |  |  |  |  |  |  |  |  |  |  |  |  |
| Cetirizine |  |  |  | 0.103 |  |  |  |  |  |  |  |  |  |  |  | 0.083 |  |  |  |  |  | 0.093 | 0.014 |
| Sotalol |  | 0.172 | 0.076 |  |  |  |  |  | 0.161 |  |  |  |  |  |  |  |  |  |  |  |  | 0.136 | 0.053 |
| Sulfamethoxazole |  | 0.348 | 0.291 |  |  | 0.030 |  | 0.154 | 0.119 | 0.118 | 0.092 | 0.093 |  |  |  | 0.033 |  |  |  |  |  | 0.142 | 0.109 |
| Tramadol |  |  |  |  |  |  |  |  |  |  |  |  |  |  |  |  |  |  |  |  | 0.200 | 0.200 |  |
| Trimethoprim |  | 0.411 | 0.213 |  |  | 0.162 | 0.360 | 0.350 | 0.238 |  |  |  |  |  |  | 0.068 |  |  |  |  | 0.178 | 0.248 | 0.117 |
| Valsartan |  |  |  |  |  |  |  |  |  |  |  |  |  |  |  |  |  |  |  | 0.048 |  | 0.048 |  |
| Venlafaxine |  | 0.388 | 0.167 |  |  |  |  |  |  |  |  |  |  |  |  | 0.055 |  |  |  |  | 0.324 | 0.234 | 0.151 |
| O-Desmethylvenlafaxine |  | 0.158 | 0.159 |  |  |  |  |  |  |  |  |  |  |  |  |  |  |  |  |  |  | 0.159 | 0.001 |

Table S5. Sources for calculating sampling rates R_Kow_ based on LogK_OW_ and Eq. 2.

|  |  |  | **Source** | | | | |
| --- | --- | --- | --- | --- | --- | --- | --- |
| **Substance** | **CAS number** | **CID** | **XLogP** | **DrugBank** | **HMDB^1^** | **HSDB^2^** | **ICSCs^3^** |
| 4-Acetamidoantipyrine | 83-15-8 | 65743 | 0 |  |  |  |  |
| 4-Formylaminoantipyrine | 1672-58-8 | 72666 | 0 |  |  |  |  |
| Amiodarone | 1951-25-3 | 2157 | 7.6 | 7.2 | 7.9 |  |  |
| Amisulpride | 71675-85-9 | 2159 | 1.5 | 1.06 | 1.1 |  |  |
| Amitriptyline | 50-48-6 | 2160 | 5 | 4.92 | 4.9 | 4.92 |  |
| Amlodipine | 88150-42-9 | 2162 | 3 | 3 | 3 | 3 |  |
| Amoxicillin | 26787-78-0 | 33613 | -2 | 0.87 |  | 0.87 |  |
| Ampicillin | 69-53-4 | 6249 | -1.1 | 1.35 | 1.35 |  |  |
| Acetanilide | 103-84-4 | 904 | 1.2 |  | 1.16 | 1.16 |  |
| Atenolol | 29122-68-7 | 2249 | 0.2 | 0.16 | 0.16 | 0.16 |  |
| Atorvastatin | 134523-00-5 | 60823 | 5 | 6.36 |  |  |  |
| Azathioprine | 446-86-6 | 2265 | 0.1 | 0.1 | 0.7 | 0.1 |  |
| Azithromycin | 83905-01-5 | 447043 | 4 | 3.03 | 4.02 | 4.02 |  |
| Beclomethasone | 4419-39-0 | 20469 | 2.2 |  |  |  |  |
| Bendroflumethiazide | 73-48-3 | 2315 | 1.2 | 1.89 | 1.7 | 1.19 |  |
| Benzotriazole | 95-14-7 | 7220 | 1 |  |  | 1.44 |  |
| Benzylpenicillin (Penicillin G) | 61-33-6 | 5904 | 1.8 | 1.83 | 1.5 | 1.83 |  |
| Bezafibrate | 41859-67-0 | 39042 | 3.8 |  |  |  |  |
| Bicalutamide | 90357-06-5 | 2375 | 2.3 | 2.5 | 2.5 |  |  |
| Bisoprolol (ß-Adrenergics) | 66722-44-9 | 2405 | 1.9 | 2.2 | 2.2 | 1.87 |  |
| Bromocriptine | 25614-03-3 | 31101 | 3.8 | 3.5 | 3.5 |  |  |
| Budesonide | 51333-22-3 | 5281004 | 2.5 | 1.914 | 1.9 | 3.2 |  |
| Buspirone | 36505-84-7 | 2477 | 2.6 | 2.63 | 2.3 |  |  |
| Dapsone | 80-08-0 | 2955 | 1 | 0.97 | 0.4 | 0.97 |  |
| Dexmedetomidine | 113775-47-6 | 5311068 | 3.1 | 2.8 | 2.8 |  |  |
| Desloratadine | 100643-71-8 | 124087 | 4.5 | 3.2 | 3.2 |  |  |
| Diatrizoate (Amidotriazotate) | 117-96-4 | 2140 | 1.8 | 3.3 | 3.3 |  |  |
| Diclofenac | 15307-86-5 | 3033 | 4.4 | 4.51 | 3.9 | 4.51 |  |
| Diltiazem | 42399-41-7 | 39186 | 3.1 | 2.8 | 2.8 | 2.7 |  |
| Doxycycline | 564-25-0 | 54671203 | -0.7 | 0.63 |  |  |  |
| Enalapril | 75847-73-3 | 5388962 | -0.1 | 0.07 | 2.1 | 0.07 |  |
| Enrofloxacin | 93106-60-6 | 71188 | -0.2 |  |  |  |  |
| Erythromycin | 114-07-8 | 12560 | 2.7 | 2.6 | 3.06 | 3.06 |  |
| Estrone | 53-16-7 | 5870 | 3.1 | 2.6 | 3.13 | 3.13 |  |
| Ethinylestradiol | 57-63-6 | 5991 | 3.7 | 3.67 | 3.67 | 3.67 |  |
| Febantel | 58306-30-2 | 1.35E+08 | 3.8 |  |  |  |  |
| Fexofenadine | 83799-24-0 | 3348 | 3 |  |  |  |  |
| Phenazone | 60-80-0 | 2206 | 0.4 | 0.38 | 0.38 |  |  |
| Fenbendazole | 43210-67-9 | 3334 | 3.6 |  |  |  |  |
| Florfenicol | 73231-34-2 | 114811 | 0.8 |  |  |  |  |
| Flubendazole | 31430-15-6 | 35802 | 2.9 |  |  |  |  |
| Fluconazole | 86386-73-4 | 3365 | 0.4 | 0.5 | 0.4 |  |  |
| Fluoxetine | 54910-89-3 | 3386 | 4 | 4.05 | 4.6 |  |  |
| Flutamide | 13311-84-7 | 3397 | 3.3 | 3.35 | 2.6 |  |  |
| Fluvastatin | 93957-54-1 | 446155 | 3.5 | 4.5 |  |  |  |
| Fluvoxamine | 54739-18-3 | 5324346 | 2.6 | 3.2 |  |  |  |
| Furosemide | 54-31-9 | 3440 | 2 | 2.03 | 2.03 | 2.03 |  |
| Gabapentin | 60142-96-3 | 3446 | -1.1 | 1.25 | -1.1 | -1.1 |  |
| Gemfibrozil | 25812-30-0 | 3463 | 3.8 | 4.387 | 3.4 |  |  |
| Glyburide (Glibenclamide) | 10238-21-8 | 3488 | 4.8 | 3.754 | 4.7 |  |  |
| Hydrochlorothiazide | 58-93-5 | 3639 | -0.1 | -0.07 | -0.07 | -0.07 |  |
| Hydrocortisone | 50-23-7 | 5754 | 1.6 | 1.61 | 1.61 | 1.61 |  |
| Ibuprofen | 15687-27-1 | 3672 | 3.5 | 3.97 | 3.97 | 3.97 |  |
| Iopamidol | 60166-93-0 | 65492 | -2.4 |  |  | -2.42 |  |
| Iopromide | 73334-07-3 | 3736 | -2.1 |  | -2.05 |  |  |
| Ipratropium | 60205-81-4 | 657309 | 2.6 | 0.038 |  |  |  |
| Irbesartan | 138402-11-6 | 3749 | 4.1 | 4.5 | 6 |  |  |
| Irinotecan | 97682-44-5 | 60838 | 3 | 3.2 | 3.2 |  |  |
| Ivermectin | 70288-86-7 | 3001671 | 3.8 |  |  |  |  |
| Ivermectin | 70288-86-7 | 6321424 | 4.1 |  |  |  |  |
| Candesartan | 139481-59-7 | 2541 | 4.1 |  | 6.1 |  |  |
| Carbamazepine | 298-46-4 | 2554 | 2.5 | 2.77 | 2.3 | 2.45 |  |
| Carvedilol | 72956-09-3 | 2585 | 4.2 | 3.8 | 3.8 | 4.19 |  |
| Quetiapine | 111974-69-7 | 5002 | 2.1 | 2.81 |  |  |  |
| Ketoconazole | 65277-42-1 | 456201 | 4.3 | 4.35 |  | 4.34 | 4.35 |
| Ketoprofen | 22071-15-4 | 3825 | 3.1 | 3.12 | 3.2 |  |  |
| Clarithromycin | 81103-11-9 | 84029 | 3.2 | 3.16 | 1.7 | 3.16 |  |
| Clenbuterol | 37148-27-9 | 2783 | 2.2 |  |  |  |  |
| Clindamycin | 18323-44-9 | 446598 | 2.2 | 2.16 |  | 2.16 |  |
| Clotrimazole | 23593-75-1 | 2812 | 5 | 6.1 |  |  |  |
| Clozapine | 5786-21-0 | 1.35E+08 | 3.1 | 3.23 | 2.7 | 3.23 |  |
| Crotamiton | 483-63-6 | 688020 | 2.8 |  |  |  |  |
| Xylometazoline | 526-36-3 | 5709 | 3.2 | 3.2 | 3.2 |  |  |
| Lamotrigine | 84057-84-1 | 3878 | 1.4 | 1.93 | 2.5 | 2.57 |  |
| Levosimendan | 141505-33-1 | 3033825 | 2.3 |  |  |  |  |
| Lidocaine | 137-58-6 | 3676 | 2.3 | 2.44 | 2.1 | 2.26 |  |
| Loratadine | 79794-75-5 | 3957 | 5.2 |  | 5.2 | 5.2 |  |
| Losartan | 114798-26-4 | 3961 | 4.3 | 1.19 | 6.1 |  |  |
| Mebendazole | 31431-39-7 | 4030 | 2.8 | 2.83 | 2.8 | 2.83 |  |
| Meropenem | 119478-56-7 | 441130 | -2.4 | -0.6 | -0.6 |  |  |
| Metaflumizone | 139968-49-3 | 9827529 | 6.2 |  |  |  |  |
| Metoprolol | 37350-58-6 | 4171 | 1.9 | 2.15 | 1.88 | 1.88 |  |
| Methotrexate | 59-05-2 | 126941 | -1.8 | -1.85 | -2.2 | -1.85 |  |
| Metronidazole | 443-48-1 | 4173 | 0 | -0.02 | -0.1 | -0.02 |  |
| Mianserin | 24219-97-4 | 4184 | 3.4 |  |  |  |  |
| Miconazole | 22916-47-8 | 4189 | 5.3 |  | 6.1 |  |  |
| Mirtazapine | 61337-67-5 | 4205 | 3.3 | 2.9 | 2.9 |  |  |
| Mometasone furoate | 83919-23-7 | 441336 | 3.9 | 4.115 |  |  |  |
| N4-Acetylsulfamethoxazole | 21312-10-7 | 65280 | 0.7 |  | 0.86 |  |  |
| Naproxen | 22204-53-1 | 156391 | 3.3 | 3.18 | 3.18 | 3.18 |  |
| N-Demethylerythromycin A | 992-62-1 | 10010092 | 2.2 |  |  |  |  |
| Nelfinavir | 159989-64-7 | 64143 | 5.7 | 6 | 6 |  |  |
| Nitenpyram | 150824-47-8 | 3034287 | 2.4 |  |  | -0.66 |  |
| Norfloxacin | 70458-96-7 | 4539 | -1 | -1.03 | 2.1 | 0.46 |  |
| O-Desmethylvenlafaxine | 93413-62-8 | 125017 | 2.6 |  |  |  |  |
| Ofloxacin (+Levofloxacin) | 82419-36-1 | 4583 | -0.4 | -0.39 | 2.1 | -0.39 |  |
| Oxazepam | 604-75-1 | 4616 | 2.2 | 2.24 | 2.8 | 2.24 |  |
| Oxytetracycline | 79-57-2 | 54675779 | -1.6 | -0.9 |  | -0.9 |  |
| Oxymetazoline | 1491-59-4 | 4636 | 2.9 | 3.4 | 3.4 |  |  |
| Paracetamol | 103-90-2 | 1983 | 0.5 | 0.46 | 0.46 | 0.46 | 0.49 |
| Paroxetine | 61869-08-7 | 43815 | 3.5 | 2.53 | 3.6 | 1.23 |  |
| Piperacillin | 66258-76-2 | 43672 | 0.5 | 0.3 | 0.3 |  |  |
| Praziquantel | 55268-74-1 | 4891 | 2.7 |  | 2.5 |  |  |
| Primidone | 125-33-7 | 4909 | 0.9 | 0.91 | 1.6 | 0.91 |  |
| Propafenone | 54063-53-5 | 4932 | 3.3 | 3.2 | 3.2 |  |  |
| Propranolol | 525-66-6 | 4946 | 3 | 3.48 | 3.48 |  |  |
| Pyrantel | 15686-83-6 | 708857 | 1.6 |  |  |  |  |
| Raloxifene | 84449-90-1 | 5035 | 6.1 | 5.2 | 5.2 |  |  |
| Ramipril | 87333-19-5 | 5362129 | 1.4 | 2.9 | 2.9 |  |  |
| Risperidone | 106266-06-2 | 5073 | 2.7 |  |  |  |  |
| Roxithromycin | 80214-83-1 | 6915744 | 3.1 | 1.7 |  |  |  |
| Salbutamol (Albuterol) | 18559-94-9 | 2083 | 0.3 | 1.4 | 1.4 | 0.64 |  |
| Salmeterol | 89365-50-4 | 5152 | 3.9 | 4.2 | 4.2 | 4.15 |  |
| Cetirizine | 83881-51-0 | 2678 | 1.7 | 2.8 |  | 1.7 |  |
| Simvastatin | 79902-63-9 | 54454 | 4.7 | 4.68 | 4.68 | 4.68 |  |
| Ciprofloxacin | 85721-33-1 | 2764 | -1.1 | 0.28 | 2.3 | 0.28 |  |
| Citalopram (+Escitalopram) | 59729-33-8 | 2771 | 3.2 | 3.76 |  |  |  |
| Sotalol | 3930-20-9 | 5253 | 0.2 |  | 1.1 |  |  |
| Sulfadiazine | 68-35-9 | 5215 | -0.1 | -0.09 | -0.2 |  |  |
| Sulfadimidine (Sulfamethazine) | 57-68-1 | 5327 | 0.3 | 0.89 | 0.89 | 0.14 |  |
| Sulfadoxine | 2447-57-6 | 17134 | 0.7 | 0.7 | 0.7 |  |  |
| Sulfaguanidine | 57-67-0 | 5324 | -0.7 |  |  |  |  |
| Sulfamerazine | 127-79-7 | 5325 | 0.1 | 0.14 | 0.14 |  |  |
| Sulfamethizole | 144-82-1 | 5328 | 0.5 | 0.54 | 0.9 | 0.54 |  |
| Sulfamethoxazole | 723-46-6 | 5329 | 0.9 | 0.89 | 0.7 | 0.89 |  |
| Cyclophosphamide | 50-18-0 | 2907 | 0.6 | 0.8 | 0.8 | 0.63 | 0.63 |
| Tamoxifen | 10540-29-1 | 2733526 | 7.1 |  | 7.1 |  |  |
| Terbutaline | 23031-25-6 | 5403 | 0.9 | 0.9 | 1.4 |  |  |
| Tetraconazole | 112281-77-3 | 80277 | 4.4 |  |  | 3.56 |  |
| Tetracycline | 60-54-8 | 54675776 | -2 | -1.3 |  | -1.37 |  |
| Tramadol | 27203-92-5 | 33741 | 2.6 | 1.34 | 2.4 |  |  |
| Trimethoprim | 738-70-5 | 5578 | 0.9 | 0.91 | 0.6 | 0.91 |  |
| Zolpidem | 82626-48-0 | 5732 | 2.5 | 3.02 |  |  |  |
| Zopiclone | 43200-80-2 | 5735 | 0.5 | 0.8 | 0.8 |  |  |
| Valsartan | 137862-53-4 | 60846 | 4.4 | 1.499 | 5.8 | 4 |  |
| Warfarin | 81-81-2 | 54678486 | 2.7 | 2.7 | 2.7 | 2.7 | 2.6 |
| Venlafaxine | 93413-69-5 | 5656 | 2.9 |  |  | 3.2 |  |
| Verapamil | 52-53-9 | 2520 | 3.8 |  | 3.79 | 3.79 |  |
| Estradiol (17ß-Estradiol) (E2) | 50-28-2 | 5757 | 4 | 4.01 | 4.01 | 4.01 |  |
| Cloxacillin | 61-72-3 | 6098 | 2.4 | 2.48 | 3 | 2.44 |  |
| Caffeine | 58-08-2 | 2519 | -0.1 | -0.07 | -0.07 | -0.07 | -0.07 |
| Methylprednisolone | 83-43-2 | 6741 | 1.9 | 1.525 | 1.5 |  |  |
| Sertraline and Norsertraline | 79617-96-2 | 68617 | 4.8 | 5.51 |  |  |  |
| Sulfathiazole | 72-14-0 | 5340 | 0.1 | 0.05 | 0.05 | 0.05 |  |
| Toremifene | 89778-26-7 | 3005573 | 7.2 | 6.8 | 6.8 |  |  |
| Triclocarban | 101-20-2 | 7547 | 5.3 |  |  | 4.342 |  |
| Entacapone | 130929-57-6 | 5281081 | 2.1 | 2.8 |  |  |  |
| 4/6-methyl-1H-benzotriazole | 136-85-6 | 8705 | 1.4 |  |  |  |  |
| Clofibric acid | 882-09-7 | 2797 | 2.6 |  |  |  |  |
| Clopidol (Methichlorpindol) | 2971-90-6 | 18087 | 2.6 |  |  |  |  |
| Propyphenazone | 479-92-5 | 3778 | 1.7 |  |  |  |  |

^1^Human Metabolome Database

^2^Hazardous Substances Data Bank; ^3^ILO-WHO International Chemical Safety Cards

Table S6. Threshold concentrations for selected substances.

| **API** | **CAS number** | **Threshold concentration [µg/L]** | **Threshold type** |
| --- | --- | --- | --- |
|  |  |  |  |
| 17α-ethinylestradiol (EE2) | 57-63-6 | 0.0000170^1)^ | AA-EQS proposal |
| 17β-estradiol (E2) | 50-28-2 | 0.000180^1)^ | AA-EQS proposal |
| Amisulpride | 71675-85-9 | 140^2)^ | PNEC |
| Amoxicillin | 26787-78-0 | 0.0780^3,7)^ | PNEC (max. MDL/MQL) |
| Azithromycin | 83905-01-5 | 0.0190^1,7)^ | AA-EQS proposal |
| Candesartan | 139481-59-7 | 1.20^4)^ | PNEC |
| Carbamazepine | 298-46-4 | 2.50^1)^  50^7)^ | AA-EQS proposal  PNEC |
| Ciprofloxacin | 85721-33-1 | 0.0890^3)^ | PNEC (max. MDL/MQL) |
| Citalopram | 59729-33-8 | 0.00635^5)^ | PNEC |
| Clarithromycin | 81103-11-9 | 0.130^1)^ | MAC-EQS proposal |
| Clindamycin | 18323-44-9 | 0.0440^6)^ | PNEC (max. MDL/MQL) |
| Clotrimazole | 23593-75-1 | 0.0200^6)^ | PNEC (max. MDL/MQL) |
| Diclofenac | 15307-86-5 | 0.0400^1)^  0.05^7)^ | AA-EQS proposal  PNEC |
| Erythromycin | 114-07-8 | 0.500^1)^ | AA-EQS proposal |
| Estrone (E1) | 53-16-7 | 0.000360^1)^ | AA-EQS proposal |
| Fluconazole | 86386-73-4 | 0.250^6)^ | PNEC (max. MDL/MQL) |
| Gualynurea | 141-83-3 | 100^6)^ | PNEC (max. MDL/MQL) |
| Hydrochlorothiazide | 58-93-5 | 56.2^4)^ | PNEC |
| Ibuprofen | 15687-27-1 | 0.140^1)^ | AA-EQS proposal |
| Irbesartan | 138402-11-6 | 700^2)^ | PNEC |
| Metformin | 657-24-9 | 156^6)^ | PNEC (max. MDL/MQL) |
| Metoprolol | 37350-58-6 | 0.100^5)^ | PNEC |
| Miconazole | 22916-47-8 | 0.200^6)^ | PNEC (max. MDL/MQL) |
| O-desmethylvenlafaxine | 93413-62-8 | 0.0600^6)^ | PNEC (max. MDL/MQL) |
| Ofloxacin | 82419-36-1 | 0.0260^6)^ | PNEC (max. MDL/MQL) |
| Primidone | 125-33-7 | 9.11^2, 7)^ | PNEC |
| Sulfamethoxazole | 723-46-6 | 0.100^6)^ | PNEC (max. MDL/MQL) |
| Triclosan | 3380-34-5 | 0.0200^1)^ | MAC-EQS proposal |
| Trimethoprim | 738-70-5 | 0.100^6, 7)^ | PNEC (max. MDL/MQL) |
| Venlafaxine | 93413-69-5 | 0.0600^6)^ | PNEC (max. MDL/MQL) |

^1)^ Proposal COM(2022a) 540 final for a directive of the European Parliament and of the Council amending Directive 2000/60/EC establishing a framework for Community action in the field of water policy, Directive 2006/118/EC on the protection of groundwater against pollution and deterioration and Directive 2008/105/EC on environmental quality standards in the field of water policy (2022/0344 (COD)). Brussels, 26.10.2022.

^2)^ Giunchi, V., Poluzzi, E. (2023). A collection of Predicted No-Effect Concentrations of human pharmaceuticals and their metabolites. bioRxiv, 2023.12.12.571257.

^3)^ EU 2020. Commission Implementing Decision (EU) 2020/1161 of 4 August 2020 establishing a watch list of substances for Union-wide monitoring in the field of water policy pursuant to Directive 2008/105/EC of the European Parliament and of the Council (notified under document number C(2020) 5205) (2020).

^4)^ Zhou, S., Di Paolo, C., Wu, X., Shao, Y., Seiler, T.-B., Hollert, H. (2019). Optimization of screening-level risk assessment and priority selection of emerging pollutants – The case of pharmaceuticals in European surface waters. *Environment International, 128*, 1-10.

^5)^ Äystö, L., Vieno, N., Fjäder, P., Mehtonen, J., Nystén, T. (2023). Hospitals and households as primary emission sources for risk-posing pharmaceuticals in municipal wastewater. *Ecotoxicology and Environmental Safety, 262*.

^6)^ Union E (2022) Commission Implementing Decision (EU) 2022/1307 of 22 July 2022: establishing a watch list of substances for Union-wide monitoring in the field of water policy pursuant to Directive 2008/105/EC. Official J Eur Union, L 197:117–121

^7)^ Finckh, S., Beckers, L.M., Busch, W., Carmona, E., Dulio, V., Kramer, L., Krauss, M., Posthuma, L., Schulze, T., Slootweg, J., von der Ohe, P.C., Brack, W. (2022). A risk based assessment approach for chemical mixtures from wastewater treatment plant effluents. Environment International, 164.
